# Molecular identification of antibiotic-resistant and virulent *Escherichia coli and Staphylococcus aureus* isolated from dogs in Southern Benin

**DOI:** 10.5455/javar.2025.l907

**Published:** 2025-05-07

**Authors:** Ayaovi Bruno Yaovi, Arpita Das, Rama N. Behera, Prudencio Comlan Sossa-Minou, Vinod Singh Bisht, Monica Yadav, Ayushi Kapoor, François Dossa, Paulin Azokpota, Souaϊbou Farougou, Lamine Baba-Moussa, Kiran Ambatipudi, Philippe Sessou

**Affiliations:** 1 Research Unit on Communicable Diseases, Polytechnic School of Abomey-Calavi, University of Abomey-Calavi, Abomey-Calavi, Benin; 2 Department of Biosciences and Bioengineering, Indian Institute of Technology Roorkee, Roorkee, India; 3 Laboratory of Food Science and Technology, University of Abomey-Calavi, Cotonou, Benin; 4 Laboratory of Biochemistry and Molecular Typing in Microbiology, Department of Biochemistry and Cell Biology, Faculty of Science and Technology, University of Abomey-Calavi, Abomey-Calavi, Benin

**Keywords:** Prevalence, Escherichia coli, Staphylococcus aureus, Dogs, Benin

## Abstract

**Objective:**

Antibiotic resistance is a worldwide health challenge. Close interaction with dogs carrying antibiotic-resistant zoonotic agents poses a risk to human health. The present study aimed to characterize antibiotic-resistant *Escherichia coli* (*E*. *coli*) and *Staphylococcus aureus* (*S*. *aureus*) isolated from dogs in Southern Benin.

**Materials and methods:**

A total of 336 swabs (112 buccal, 112 nasal, and 112 rectal) from 112 dogs living in the communes of Abomey-Calavi and Cotonou were analyzed for *E*. *coli* and *S*. *aureus* presence. Bacterial isolates were tested for antibiotic (penicillins, tetracyclines, aminoglycosides, cephalosporins, sulfonamides, and macrolides) susceptibility using the disc diffusion method, and antibiotic-resistant strains were characterized by the polymerase chain reaction method.

**Results:**

A 41.07% and 20.53% of dogs harbored *E*. *coli* and *S*. *aureus*, respectively. *Escherichia coli* and *S*. *aureus* isolates showed resistance to penicillin (100% and 81.48%), tetracycline (44.64% and 59.26%), and other antimicrobials tested. *Escherichia coli* isolates harbored resistance genes *bla*TEM (63.46%), *tetA* (62.50%), and *strA-strB* (55.56%). *tetK* (100%), *tetM* (100%), and *bla*Z (82.61%) were present in *S*. *aureus* isolates. *Escherichia coli* strains harbored virulence genes *fimH* (61.54%), *kpsMTII* (26.92%), *fyuA* (19.23%), and *eae* (1.92%), whereas 20.83% of *S*. *aureus* strains harbored *pvl* and *fnbA*.

**Conclusion:**

The results of the current study reveal the urgent need for stricter controls on antibiotic use. Implementing guidelines, responsible prescribing, and increasing public awareness are crucial steps to address this problem.

## Introduction

The emergence and re-emergence of zoonoses represent significant global health challenges. Zoonoses are infectious diseases that can be transmitted naturally from vertebrate animals to humans or from humans to vertebrate animals [1]. These diseases are caused by a broad range of bacteria, viruses, fungi, protozoa, parasites, and other pathogens. The pathogenic power of these microorganisms is due to the virulence factors they produce in the host body under the action of various virulence genes [2]. Given the diversity and complexity of symptoms associated with zoonotic infections, the diagnosis remains challenging due to the poor skill set of animal and human health workers and limited resources. The frequent use and/or misuse of antibiotics has led pathogens to develop different antibiotic-resistant mechanisms, including efflux pumps, alteration of the drug target site, enzymatic inactivation of the antimicrobial agent, and sequestration of the antimicrobial agent [3]. These mechanisms are encoded by several genes that can be transmitted horizontally or vertically [4].

Nowadays, the issue of antimicrobial resistance (AMR) further complicates the treatment protocols for numerous zoonotic diseases. The direct consequences of infection with antibiotic-resistant microorganisms can be serious: longer duration of illness, higher mortality, prolonged hospitalization, weakened protection during surgery or other medical procedures, and increased costs [5]. The indirect consequences of AMR include economic losses associated with decreased productivity due to the disease and higher treatment costs [5]. A study revealed that resistance to anti-infectives could be responsible for well over 10 million deaths a year and become the leading cause by 2050, entailing an economic cost of US$100 billion if precautions are not taken [6].

The involvement of animals in transmitting many infections to humans has been widely demonstrated. Dogs are beloved pets for many of their owners. However, they are recognized as reservoirs of zoonotic agents, including *Staphylococcus aureus* and *Escherichia coli* strains [7,8]. *Staphylococcus aureus* is one of the main pathogens causing a wide range of infections in humans and animals, such as bacteremia, skin and tissue infections, pneumonia, urinary tract infections, pyoderma, abscesses, and wound infections [9]. *Escherichia coli* strains were associated with diarrhea, hemorrhagic colitis, hemolytic uremic syndrome in humans, dysentery, chronic inflammatory bowel disease, neonatal meningitis, and so on [2]. Many studies reported antibiotic-resistant *S*. *aureus* and *E*. *coli* strains in dogs. However, the close physical contact often observed between dogs and their owners reveals the risk incurred by all those who come into contact with dogs harboring antibiotic-resistant pathogens [10]. Faced with this risk, dogs must be monitored frequently to prevent human contamination and AMR as early as possible.

In Benin, and specifically in the municipalities of Abomey-Calavi and Cotonou, the number of households owning dogs is growing. However, no study has provided information on the prevalence of *S*. *aureus* and *E*. *coli* strains harbored by dogs or their susceptibility to standard antibiotics. This lack of information is a limitation to bacterial zoonosis management, particularly those caused by these bacterial species, and the risks of antibiotic resistance.

The present research, therefore, focused on characterizing antibiotic-resistant zoonotic pathogens such as *E*. *coli* and *S*. *aureus* in dogs in Abomey-Calavi and Cotonou municipalities, southern Benin, by determining their antibiotic susceptibility and the prevalence of resistance and virulence genes that they harbor, to fight the infections they cause and the antibiotic resistance phenomenon observed in dogs within these municipalities.

## Materials and Methods

### Ethical approval

The present study was approved by the Ethical Committee of Research Unit on Communicable Diseases (URMAT in French) of the Polytechnic School of Abomy-Calavi of the University of Abomey-Calavi (N°004/EPAC/LARBA/ URMAT/CE/R).

### Collection of samples

The current study was carried out from May 2022 to February 2023 in Abomey-Calavi and Cotonou municipalities in southern Benin. It was approved by the Ethical Committee of the Research Unit on Communicable Diseases (URMAT in French) of the Polytechnic School of Abomy-Calavi of the University of Abomey-Calavi (N°004/ EPAC/LARBA/URMAT/CE/R). Using a sterile swab (Meus Srl, Piove di Sacco, Italy) soaked in sterile distilled water, a total of 336 samples, including 112 buccal mucosa, 112 nasal, and 112 rectal, were taken from 112 dogs in the two municipalities. Samples were kept cool in a dry ice cooler and then transferred to the laboratory within 45 min for microbiological analysis.

### Isolation and identification of zoonotic bacteria

Isolation of *E*. *coli* strains was carried out using Rapid’ *E*. *coli* 2 agar and Eosin Methylene Blue (EMB agar) medium [11]. Each sample was inoculated onto the surface of Rapid’ *E*. *coli* 2 agar (Bio-Rad, Marnes, France) Petri dishes. The plates were incubated at 37°C for 24 h under aerobic conditions. After incubation, plates with purple to pink colonies were retained, and one to three colonies were streaked onto Rapid’ *E*. *coli* 2 agar and incubated at 37°C for 24 h. One purified colony was streaked onto EMB agar (Himedia, Dindori, India) and incubated further at 37°C for 24 h. The purple colonies, which are *E*. *coli* characteristics on the agar EMB, were grown on nutrient agar, and each isolate was tested for indole, catalase, and oxidase production [12]. Isolates producing indole, catalase, and not oxidase were retained in this study.

*Staphylococcus aureus* strains were isolated following the procedure described by Youn et al. [13]. The pre-enrichment inoculum was inoculated directly onto Baird-Parker agar (Oxoid, Basingstoke, UK) and incubated at 37°C for 24 to 48 h. Plates showing black colonies surrounded by a clear halo were retained as positive for *S*. *aureus*. Presumptive *S*. *aureus* colonies were purified by successive subculturing on Baird-Parker agar. After purification, *S*. *aureus* colonies grown on nutrient agar were subjected to coagulase testing using rabbit plasma and motility, H_2_S, and indole production tests on a sulfide-indole-motility (SIM) medium. Coagulase-positive, immobile isolates producing neither H₂S nor indole were used further.

### Antibiotic susceptibility testing for zoonotic bacteria

To test the antibiotic susceptibility of *E*. *coli* and *S*. *aureus*, the disc diffusion method on Mueller-Hinton agar (Oxoid, Basingstoke, UK) was used, considering the recommendations of the Antibiogram Committee of the French Society for Microbiology [14]. Bacterial inoculum was prepared by suspending one or two colonies of each strain in 5 ml of Mueller-Hinton broth, homogenized to the 0.5 McFarland scale. On Mueller-Hinton agar, bacterial inoculum was swabbed, and antibiotic discs from different families were placed with sterile forceps. Nine antibiotics from six antimicrobial classes were tested on *E*. *coli* strains, including penicillins (penicillin G; amoxicillin-clavulanic acid 30 μg), tetracyclines (tetracycline 30 μg), aminoglycosides (gentamicin 10 μg, streptomycin 10 μg), phenicols (chloramphenicol 30 μg), cephalosporins (ceftazidime 30 μg, cefotaxime 30 μg), and sulfonamides (sulfamethoxazole-trimethoprim or cotrimoxazole 25 μg) (Oxoid, Basingstoke, UK). In addition to these antibiotics, erythromycin (15 μg) was tested on *S*. *aureus* strains. After 15 min of antibiotic placement on Mueller-Hinton agar, plates were incubated at 37°C for 16–24 h. The inhibition zones’ diameters around the antibiotic discs were measured, and results were interpreted as Susceptible, Intermediate, or Resistant according to CASFM and EUCAST criteria [14]. *S*. *aureus* ATCC 25923 and *E*. *coli* ATCC 25922 were used as control strains for quality assurance.

### Detection of resistance and virulence genes

The process for detecting resistance and virulence genes in antibiotic-resistant strains of zoonotic bacteria involved several steps:

### DNA extraction

*Escherichia coli*; *Staphylococcus aureus* DNA extraction was performed by modifying the Phenol-Chloroform-Isoamyl (PCI) method, followed by Wright et al. [15]. The genomic DNA extraction kit (SRL, BioLitTM, India) was used. First, 2 ml of 16 h bacterial inoculum prepared from two or three colonies in 2 ml brain heart infusion broth was transferred to a 2 ml Eppendorf tube and centrifuged at 7500 rpm for 10 min. The supernatant was discarded, and the pellet was resuspended with 80 ml of SE buffer containing RNase. After vortexing, the contents were transferred to a 1.5 ml Eppendorf tube. Then, 12 ml of 10% sodium dodecyl sulfate (SDS) solution was added to the contents of the tube, which was then incubated at 37°C for 10 min. Next, 3 ml of potassium chloride (KCl) solution was thoroughly mixed with the contents. Furthermore, 18 ml of saturated phenol buffer and 18 ml of chloroform-isoamyl (24:1) were mixed with the tube contents. The mixture was vortexed for 30 sec, left to stand for 2 min, and vortexed again for 30 sec. Centrifugation was performed at 12,000 rpm for 15 min, and 100 ml of the upper aqueous phase was transferred to a sterile 1.5 ml Eppendorf tube.

Water bath heating was performed at 52°C for 10 min, and 100 ml of the DNA precipitation solution (−20°C, 100% ethanol) was gently mixed by inversion and added. Mixing was performed by inverting the tube and incubating at room temperature in the dark for 10 min. Centrifugation at 12,000 rpm for 20 min was then performed, and the supernatant was discarded. Furthermore, 100 ml of the wash solution was added to the tube contents and centrifuged at 12,000 rpm for 5 min. The supernatant was discarded, and the pellet was dried for 3–5 min at room temperature. The DNA pellet was resuspended with 30 ml TE buffer and stored at 40°C for 24 h before use. To confirm bacterial DNA’s presence and concentration, 2 μl of the product was run on a 1% agarose gel.

### PCR amplification of virulence and resistance genes

A total volume of 12.5 μl was used for the polymerase chain reaction (PCR) amplification, which contained 0.3 μl DNA template, 6.25 μl Master Mix (Emerald, TaKaRa, Japan), 0.5 μl (10 pmol) of primer forward, and 0.5 μl (10 pmol) of primer reverse, and then 4.95 μl of nuclease-free water (dH_2_O). The amplification cycle was repeated 30 times. To check amplification results, 3 μl of PCR products were resolved on a 1% agarose gel prepared with TAE buffer and stained with ethidium bromide, and 2 μl of a GeneRuler 1kb Plus DNA Ladder molecular weight marker (Thermo Scientific™) was used to estimate amplicon size. Electrophoresis was performed at 100 volts for 25 min. The DNA bands were visualized using the ChemiDoc MP Imaging System (Bio-Rad, USA). The primer sequences and the PCR conditions used to detect resistance and virulence genes of *E*. *coli* and *S*. *aureus* are listed in the supplementary tables, Tables 1 and 2.

**Table 1.** Table 1. Resistance genes tested for *E. coli* and *Staphylococcus aureus.*

Antibiotics	Resistance genes		Primer sequences (5’-3’)	Amplicon size (bp) Heating	Amplification conditions				References
					Denaturation	Annealing	Extension		
Penicillin	*blaTEM*	F	AGTGCTGCCATAACCATGAGTG	431	94°, 5 min	94°, 1 min	61°, 1 min	72°, 1 min;72°, 5 min	[42]
		R	CTGACTCCCCGTCGTGTAGATA						
Tetracycline	*tetA*	F	GTAATTCTGAGCACTGTCGC	937	95°, 5 min	95°, 30 sec	62°C, 30 sec	72° for 45 sec and 72° for 7 min	[43]
		R	CTGTCCTGGACAACATTGCTT						
	*tetB*	F	CTCAGTATTCCAAGCCTTTG	416	95°, 5 min	95°, 30 sec	57°C, 30 sec	72°, 20 sec; 72°, 7 min	
		R	CTAAGCACTTGTCTCCTGTT						
Streptomycin	*strA-strB*	F	CCAATCGCAGATAGAAGGCAAG	580	94°C, 10 min	94°C, 1 min	65°C, 30 sec	72°C, 1 min;72°C, 10 min	[44]
		R	ATCAACTGGCAGGAGGAACAGG						
Penicillin	*blaZ*	F	ACTTCAACACCTGCTGCTTTC	173	94 °C, 3 min	94 °C, 30 sec	49 °C, 30 sec	72 °C, 1 min;72 °C, 8 min	[13]
		R	TGACCACTTTTATCAGCAACC						
Tetracyclins	*tetK*	F	TTAGGTGAAGGGTTAGGTCC	718	95°C, 5 min	95°C, 30 sec	55°C, 30 sec	72°C, 30 sec;72°C; 7 min	[13]
		R	GCAAACTCATTCCAGAAGCA						
	*tetM*	F	GTTAAATAGTGTTCTTGGAG	647	95°C, 5 min	95°C, 30 sec	55°C, 30 sec	72°C, 30 sec;72°C, 7 min	[13]
		R	CTAAGATATGGCTCTAACAA						
Methicillin	*mecA*	F	AAAATCGATGGTAAAGGTTGGC	532	94°C, 3 min	94°C, 30 sec	55°C, 30 sec	72°C, 30 sec;72°C, 4 min	[13]
		R	AGTTCTGCAGTACCGGATTTGC						

**Table 2.** Table 2. Virulence genes tested for *E. coli* and *Staphylococcus aureus*.

Virulence gene function	Virulence gene		Primer sequences (5’-3’)	Amplicon size (bp) Heating	Amplification conditions				References
					Denaturation	Annealing	Extension		
Escherichia coli									
Shigatoxins	stx 1	F	CAGTTAATGTGGTGGCGAAGG	348	94°C, 5 min	94°C, 1.5 min	64°C, 1.5 min	72°C, 1.5 min;72°C, 7 min	[45]
		R	CACCAGACAATGTAACCGCTG						
	stx 2	F	ATCCTATTCCCGGGAGTTTACG	584	94°C, 5 min	94°C, 1.5 min	64°C, 1.5 min	72°C, 1.5 min;72°C, 7 min	[45]
		R	GCGTCATCGTATACACAGGAGC						
Attachment	eae	F	AGGCTTCGTCACAGTTG	570	94°C, 2 min	94°C, 30 sec	50°C, 30 sec	72°C, 30sec; 72°C, 7 min	[46]
		R	CCATCGTCACCAGAGGA						
Adhesins	fimH	F	TGCAGAACGGATAAGCCGTGG	508	95°C, 12 min	94°C, 30sec	63°C, 30sec	68°C, 3 min; 72°C, 10 min	[47]
		R	GCAGTCACCTGCCCTCCGGTA						
Capsule	kpsMT II	F	GCGCATTTGCTGATACTGTTG	272	95°C, 12 min	94°C, 30 sec	63°C, 30 sec	68°C, 3 min; 72°C, 10 min	[47]
		R	CATCCAGACGATAAGCATGAGCA						
Siderophores	fyuA	F	TGATTAACCCCGCGACGGGAA	880	95°C, 12 min	94°C, 30 sec	63°C, 30 sec	68°C, 3 min; 72°C, 10 min	[47]
		R	CGCAGTAGGCACGATGTTGTA						
Staphylococcus aureus									
Panton Valentin leukocidin	pvl	F	ATCATTAGGTAAAATGTCTGGACATGATCCA	433	95°C, 5 min	94°C, 1 min	55°C, 1 min	72°C, 1 min; 72°C, 10 min	[48]
		R	GCATCAASTGTATTGGATAGCAAAAGC						
Enterotoxin	sea	F	GAAAAAAGTCTGAATTGCAGGGAACA	560	95°C, 5 min	94°C, 1 min	55°C, 1 min	72°C, 1 min; 72°C, 10 min	[48]
		R	CAAATAAATCGTAATTAACCGAAGGTTC						
	seb	F	ATTCTATTAAGGACACTAAGTTAGGGA	404	95°C, 5 min	94°C, 1 min	55°C, 1 min	72°C, 1 min; 72°C, 10 min	[48]
		R	ATCCCGTTTCATAAGGCGAGT						
	sec	F	GTAAAGTTACAGGTGGCAAAACTTG	297	95°C, 5 min	94°C, 1 min	55°C, 1 min	72°C, 1 min; 72°C, 10 min	[48]
		R	CATATCATACCAAAAAGTATTGCCGT						
Exfoliation	eta	F	CGCTGCGGACATTCCTACATGG	676	94°C, 5 min	94°C, 30 sec	57°C, 30 sec	72°C, 45 sec; 72°C, 10 min	[49]
		R	TACATGCCCGCCACTTGCTTGT						
	etb	F	CAGATAAAGAGCTTTATACACACATTAC	612	95°C, 5 min	94°C, 1 min	55°C, 1 min	72°C, 1 min; 72°C, 10 min	[48]
		R	AGTGAACTTATCTTTCTATTGAAAAACACTC						
Hemolysin	*hla*	F	CTGATTACTATCCAAGAAATTCGATTG	209	95°C, 5 min	94°C, 1 min	55°C, 1 min	72°C, 1 min;72°C, 10 min	[48]
		R	CTTTCCAGCCTACTTTTTTATCAGT						
	hlb	F	GTGCACTTACTGACAATAGTGC	309	95°C, 5 min	94°C, 1 min	55°C, 1 min	72°C, 1 min;72°C, 10 min	[48]
		R	GTTGATGAGTAGCTACCTTCAGT						
Adhesin	*fnbA*	F	GTGAAGTTTTAGAAGGTGGAAAGATTAG	643	94°C, 5 min	94°C, 30 sec	57°C, 30 sec	72°C, 40 sec; 72°C, 10 min	[49]
		R	GCTCTTGTAAGACCATTTTTCTTCAC						
	fnbB	F	GTAACAGCTAATGGTCGAATTGATACT	524	94°C, 5 min	94°C, 30 sec	57°C, 30 sec	72°C, 35 sec;72°C, 10 min	[49]
		R	CAAGTTCGATAGGAGTACTATGTTC						

### Partial genome sequencing and bioinformatics analysis

Amplification of the gene *fimH* for the five *E*. *coli* strains harboring the genes *bla*TEM, *tetA*, and *strA-strB*, and of the gene *tetK* for four *S*. *aureus* strains harboring *blaZ* and *tetK* was performed. To ensure accurate amplification of the target DNA sequences, the Phusion enzyme was used during the PCR process due to its high fidelity and robustness. A 50 μl reaction mixture consisting of 10 μl of HF buffer, 4 μl of dNTPs, 1 μl of each primer (10 pmol), 3 μl of DNA template, 0.4 μl of Phusion enzyme, and 30.6 μl of nuclease-free water (dH_2_O) had been made for amplification of each gene. The amplification conditions applied were 98°C for 1 min for heating, 98°C for 30 sec for denaturation, 72°C for 1 min for initial extension, and 72°C for 7 min for final extension. Hybridization was performed according to the temperature of each primer for 30 sec, and the cycle was repeated 35 times. Amplification quality was checked on the agarose gel by electrophoresis. Amplification products were then purified using the PCR Purification Kit (FavorPrepTM, FavorGen Biotech Co.) and sent to the Eurofins department (Eurofins Genomics, India) for partial sequencing of each target gene. The raw sequences were checked for quality and then assembled using BioEdit software. Using the BLAST program, the assembled sequences were compared for homology with the GenBank sequence database (National Center for Biotechnology Information, USA) [16]. An identity of 99.90% was considered the minimum acceptable to determine that the sequence obtained for each isolate corresponds to the bacterial strain studied. The assembled sequences were also used to construct phylogenetic trees, using MEGA 11 software [17]. The Neighbor-Joining method was used to infer evolutionary history [17]. To represent the evolutionary history of the taxa analyzed, the bootstrap consensus tree inferred from 1,000 replicates was taken [17]. Branches corresponding to partitions reproduced in less than 50% of bootstrap replicates are collapsed. The percentage of replicate trees in which the associated taxa clustered together in the bootstrap test (1000 replicates) is shown next to the branches. The maximum composite likelihood method was used to compute the evolutionary distances [17]. All positions containing gaps and missing data were eliminated (complete deletion option).

### Statistical analysis

*Escherichia coli* and *Staphylococcus aureus* isolation, antibiotic susceptibility testing, and resistance and virulence gene detection data were entered into an Excel spreadsheet, coded, and then subjected to statistical analysis using R 4.1.2 software. The prevalence of each zoonotic bacterium was determined according to variables such as municipality, sex, age, breed, health status, housing mode, antibiotic therapy, and dog sample types. The prevalence of antibiotic-resistant isolates, multidrug-resistant (MDR) isolates, resistance, and virulence genes was also determined. MDR strains are strains that have acquired resistance to at least one agent in three or more antimicrobial categories [18]. The *prop.test* function determined prevalences and association links between prevalences and the variables studied. For a value of *p* < 0.05, the link between prevalence and the variable studied was reported as statistically significant. In contrast, a value of *p* ≥ 0.05 indicated a statistically insignificant link between prevalence and the variable concerned.

## Results

### Characteristics of dogs

Dog characteristics included in this study are presented in Table 3. Out of 112 dogs investigated, 57.14% came from Abomey-Calavi municipality and 42.86% from Cotonou. 58.93% of the dogs were males, and 41.07% were females. The dog breeds involved in the study were mainly German shepherds (40.18%), local breeds (12.50%), poodles (12.50%), boerbulls (8.93%), rottweilers (5.36%), Swiss shepherds (1.79%), Caucasian shepherds (1.79%), Malinois shepherds (1.79%), and bichons (0.89%). Mixedbreed dogs represented 14.28% of the dogs sampled. 77.68% of the dogs were housed in cages, while 22.32% were free-ranging in households. In addition, 95.54% of the dogs were healthy, and 4.46% showed signs of disease (appetite loss, diarrhea, weight loss). 75% of the dogs in this study had undergone antibiotic therapy at least once, but 25% of the dogs had never undergone antibiotic therapy before sampling.

**Table 3.** Table 3. Characteristics of dogs.

Variables	Number of dogs	Percentage (%)
Municipalities		
Abomey-Calavi	64	57.14
Cotonou	48	42.86
Sex		
Male	66	58.93
Female	46	41.07
Age		
< 3	32	28.57
> 3	80	71.43
Breed		
Local	14	12.50
Mixed-breed	16	14.28
German shepherds	45	40.18
Swiss shepherds	2	1.79
Caucasian shepherds	2	1.79
Malinois shepherds	2	1.79
Boerbulls	10	8.93
Rottweilers	6	5.36
Poodles	14	12.50
Bichons	1	0.89
Housing mode		
In cage	87	77.68
Free-ranging	25	22.32
Health status		
Apparently healthy	107	95.54
Sick	5	4.46
Antibiotic therapy At least once	84	75.00
Never	28	25.00

### Prevalence of zoonotic bacteria isolated from dogs

**Fig. 1.**
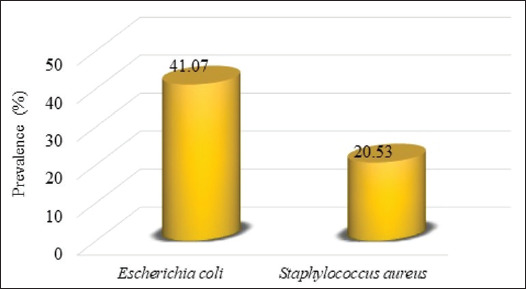
Figure 1. Overall prevalence of zoonotic bacteria isolated from dogs.

The overall *E*. *coli* prevalence (41.07%, 46/112) was higher than that of *S*. *aureus* (20.53%, 23/112) (Fig. 1). The prevalence of each bacterial isolate according to the variables is shown in Table 4.

**Table 4.** Table 4. Prevalence of zoonotic bacteria isolated from dogs.

Variables	Number of dogs	*E. coli*		*S. aureus*	
		n (%)	*p*-value	n (%)	*p*-value
Municipalities					
Abomey-Calavi	64	27 (42.18)	0.781	13 (20.31)	0.946
Cotonou	48	19 (39.58)		10 (20.83)	
Sex					
Male	66	27 (40.91)	0.966	11 (16.67)	0.224
Female	46	19 (41.30)		12 (26.09)	
Age					
< 3	32	8 (25.00)	0.028	6 (18.75)	0.767
> 3	80	38 (47.5)		17 (21.25)	
Breed					
Local	14	4 (28.57)	0.463	2 (14.29)	0.128
Mixed-breed	16	6 (37.5)		7 (43.75)	
German shepherds	45	21 (46.67)		7 (15.56)	
Swiss shepherds	2	1 (50.00)		1 (50.00)	
Caucasian shepherds	2	2 (100)		0 (0.00)	
Malinois shepherds	2	0 (0.00)		0 (0.00)	
Boerbulls	10	5 (50.00)		2 (20.00)	
Rottweilers	6	2 (33.33)		0 (0.00)	
Poodles	14	4 (28.57)		3 (21.43)	
Bichons	1	1 (100)		1 (100)	
Housing mode					
In cage	87	37 (42.53)	0.558	17 (19.54)	0.626
Free-ranging	25	9 (36.00)		6 (24.00)	
Health status					
Apparently healthy	107	41 (38.32)	0.006	21 (19.63)	0.270
Sick	5	5 (100)		2 (40.00)	
Antibiotic therapy					
At least once	84	39 (46.43)	0.045	21 (25.00)	0.042
Never	28	7 (25.00)		2 (7.14)	
Total	112	46 (41.07)		23 (20.53)	
Sample types					
Buccal	112	11 (9.82)	0.000	10 (8.93)	0.207
Nasal	112	6 (5.36)		12 (10.71)	
Rectal	112	41 (36.61)		5 (4.46)	
Total	336	58 (17.26)		27 (8.04)	

n: number of dogs in which the strains were detected; %: prevalence.

The prevalence of *E*. *coli* isolated was significantly higher in dogs over 3 years of age than in dogs under 3 years of age (*p* < 0.05). The *E*. *coli* strains were significantly Figure 1. Overall prevalence of zoonotic bacteria isolated from dogs. more frequent in sick dogs than in apparently healthy dogs (*p* < 0.05). This bacterium was more prevalent in dogs that had undergone antibiotic therapy at least once than in those that had never undergone it. However, *E*. *coli* prevalence did not vary according to municipality, sex, or housing mode. According to sample type, out of 336 swabs, *E. coli* prevalence was 17.26%. *Escherichia coli* prevalence was significantly greater in rectal swabs (36.61%) than the buccal (9.82%) and nasal swabs (5.36%) (*p* < 0.05).

As for *S*. *aureus* isolates, the prevalence was significantly higher in dogs that had undergone antibiotic therapy at least once than in dogs that had never undergone antibiotic therapy before (*p* < 0.05). Considering sample type, the prevalence of *S*. *aureus* was 10.71% for nasal swabs, 8.93% for buccal swabs, and 4.46% for rectal swabs. Additionally, *S. aureus* prevalence did not vary significantly concerning the other variables studied (*p* > 0.05).

### Antibiotic susceptibility profile of zoonotic bacteria isolated from dogs

**Fig. 2.**
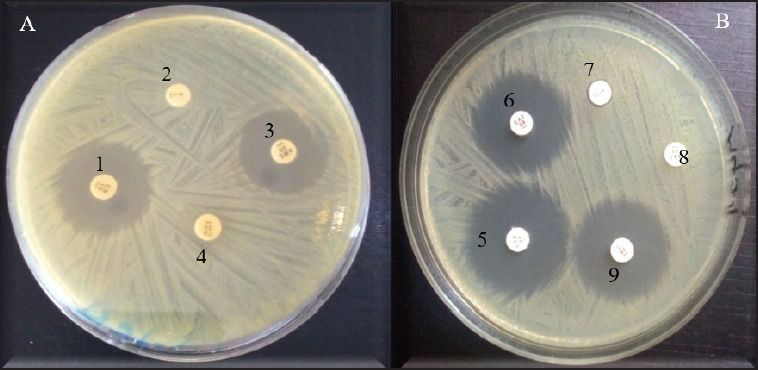
Figure 2. Antibiotic susceptibility profile of **E*. *coli** strains. (A) 1: gentamicin (CN), 2: penicillin G (P), 3: amoxicillin-clavulanic acid (AMC), 4: tetracycline (TE), (B) *5 :* cefotaxime (CTX), 6 : ceftazidime (CAZ), 7 : streptomycin (S), 8 : cotrimoxazole (SXT), 9 : chloramphenicol.

The antibiotic resistance profile of *E*. *coli* and *S*. *aureus* isolated from dogs is shown in Table 5. Fifty-six *E*. *coli* isolates tested were resistant to penicillin (100%), but less than half were resistant to tetracycline (44.64%), cotrimoxazole (19.64%), streptomycin (16.07%), amoxicillin-clavulanic acid (8.93%), gentamicin (7.14%), chloramphenicol (3.57%), ceftazidime (1.79%), and cefotaxime (1.79%) (Fig. 2). However, the prevalence of *E. coli* resistant to tetracycline, streptomycin, chloramphenicol, and cotrimoxazole did not vary significantly according to the variables studied, while the prevalence of *E. coli* resistant to amoxicillin-clavulanic acid varied according to the health status, that of *E. coli* isolates resistant to gentamicin varied according to the breed (*p* < 0.05), and that of ceftazidime and cefotaxime-resistant *E. coli* was significantly higher in nasal swabs than in buccal and rectal swabs (*p* < 0.05) (Table 6).

**Table 5.** Table 5. Antibiotic susceptibility profile of isolated zoonotic bacteria.

Antibiotic families	Antibiotics	*E*. *coli* (56)			*S*. *aureus* (27)		
		R (%)	I (%)	S (%)	R (%)	I (%)	S (%)
Penicillins	Penicillin G	56 (100)	0 (0.00)	0 (0.00)	22 (81.48)	0 (0.00)	5 (18.52)
	Amoxicillin-clavulanic acid	5 (8.93)	2(3.57)	49 (87.50)	0 (0.00)	0 (0.00)	27 (100)
Tetracyclines	Tetracycline	25 (44.64)	0 (0.00)	31 (55.36)	16 (59.26)	0 (0.00)	11 (40.74)
Aminoglycosides	Gentamicin	4 (7.14)	0 (0.00)	52 (92.86)	1 (3.70)	0 (0.00)	26 (96.30)
	Streptomycin	9 (16.07)	10 (17.86)	37 (66.07)	6 (22.22)	2 (7.41)	19 (70.37)
Macrolides	Erythromycin	–	–	–	5 (18.52)	0 (0.00)	22 (81.48)
Phenicols	Chloramphenicol	2 (3.57)	0 (0.00)	54 (96.43)	4 (14.81)	0 (0.00)	23 (85.19)
Cephalosporins	Ceftazidime	1 (1.79)	0 (0.00)	55 (98.21)	6 (22.22)	10 (37.04)	11 (40.74)
	Cefotaxime	1 (1.79)	0 (0.00)	55 (98.21)	1 (3.70)	6 (22.22)	20 (74.07)
Sulfonamides	Cotrimoxazole	11 (19.64)	0 (0.00)	45 (80.36)	3 (11.11)	0 (0.00)	24 (88.89)
MDR		16 (28.57)	–	–	9 (33.33)	–	–

R: number of isolates resistant to the antibiotic; I: number of isolates with intermediate resistance to the antibiotic; S: number of isolates susceptible to the antibiotic; %: prevalence; MDR: multidrug-resistant isolates.

**Table 6.** Table 6. Prevalence of resistant **E*. *coli** strains according to the variables.

Variables		Penicillin G		Amoxicillinclavulanic acid		Tetracycline		Gentamicin		Streptomycin		Chloramphenicol		Ceftazidime		Cefotaxime		Cotrimoxazole	
	*N*	*n* (%)	*p*-value	*n* (%)	*p*-value	*n* (%)	*p*-value	*n* (%)	*p*-value	*n* (%)	*p*-value	*n* (%)	*p*-value	*n* (%)	*p*-value	*n* (%)	*p*-value	*n* (%)	*p*-value
Municipalities																			
Abomey-Calavi	34	34 (100)	–	3 (8.82)	0.972	15 (44.12)	0.921	2 (5.88)	0.648	5 (14.71)	0.729	1 (2.94)	0.752	1 (2.94)	0.417	1 (2.94)	0.417	8 (23.53)	0.362
Cotonou	22	22 (100)		2 (9.09)		10 (45.45)		2 (9.09)		4 (18.18)		1 (4.55)		0 (0)		0 (0)		3 (13.64)	
Sex																			
Male	34	34 (100)	–	2 (5.88)	0.320	15 (44.12)	0.921	2 (5.88)	0.648	6 (17.65)	0.689	1 (2.94)	0.752	1 (2.94)	0.417	1 (2.94)	0.417	8 (23.53)	0.362
Female	22	22 (100)		3 (13.64)		10 (45.45)		2 (9.09)		3 (13.64)		1 (4.55)		0 (0)		0 (0)		3 (13.64)	
Age																			
< 3	12	12 (100)	–	1 (8.33)	0.935	7 (58.33)	0.281	2 (16.67)	0.148	2 (16.67)	0.949	0 (0)	0.452	0 (0)	0.598	0 (0)	0.598	2 (16.67)	0.769
> 3	44	44 (100)		4 (9.09)		18 (40.91)		2 (4.55)		7 (15.91)		2 (4.55)		1 (2.27)		1 (2.27)		9 (20.45)	
Breed																			
Local	4	4 (100)	–	0 (0)	0.960	2 (50)	0.284	0 (0)	0.047	1 (25)	0.374	0 (0)	0.498	0 (0)	0.996	0 (0)	0.996	0 (0)	0.128
Mixed-breed	6	6 (100)		1 (16.67)		5 (83.33)		0 (0)		3 (50)		0 (0)		0 (0)		0 (0)		3 (50)	
German shepherds	25	25 (100)		2 (8.00)		9 (36)		0 (0)		3 (12)		0 (0)		1 (4)		1 (4)		4 (16)	
Swiss shepherds	1	1 (100)		0 (0)		0 (0)		0 (0)		0 (0)		0 (0)		0 (0)		0 (0)		0 (0)	
Caucasian shepherds	2	2 (100)		0 (0)		0 (0)		1 (50)		0 (0)		0 (0)		0 (0)		0 (0)		0 (0)	
Malinois shepherds	–	–		–		–		–		–		–		–		–		–	
Boerbulls	8	8 (100)		1 (12.50)		5 (62.50)		1 (12.50)		2 (25)		1 (12.50)		0 (0)		0 (0)		4 (50)	
Rottweilers	2	2 (100)		0 (0)		1 (50)		1 (50)		0 (0)		0 (0)		0 (0)		0 (0)		0 (0)	
Poodles	5	5 (100)		1 (20)		1 (20)		1 (20)		0 (0)		1 (20)		0 (0)		0 (0)		0 (0)	
Bichons	3	3 (100)		0 (0)		2 (66.67)		0 (0)		0 (0)		0 (0)		0 (0)		0 (0)		0 (0)	
Health status																			
Apparently healthy	50	50 (100)	–	3 (6)	0.026	22 (44)	0.78	4 (8)	0.472	8 (16)	0.966	1 (2)	0.067	1 (2)	0.726	1 (2)	0.726	9 (18)	0.371
Sick	6	6 (100)		2 (33.33)		3 (50)		0 (0)		1 (16.67)		1 (16.67)		0 (0)		0 (0)		2 (33.33)	
Housing mode																			
In cage	45	45 (100)	–	4 (8.89)	0.983	21 (46.67)	0.537	3 (6.67)	0.779	8 (17.78)	0.481	2 (4.44)	0.476	1 (2.22)	0.617	1 (2.22)	0.617	10 (22.22)	0.325
Free-ranging	11	11 (100)		1 (9.09)		4 (36.36)		1 (9.09)		1 (9.09)		0 (0)		0 (0)		0 (0)		1 (9.09)	
Antibiotic therapy																			
At least once	52	52 (100)	–	5 (9.62)	0.515	22 (42.31)	0.205	3 (5.77)	0.150	8 (15.38)	0.613	2 (3.85)	0.689	1 (1.92)	0.779	1 (1.92)	0.779	10 (19.23)	0.779
Never	4	4 (100)		0 (0)		3 (75)		1 (25)		1 (25)		0 (0)		0 (0)		0 (0)		1 (25)	
Sample types																			
Buccal	11	11 (100)	–	1 (9.09)	0.714	4 (36.36)	0.814	1 (9.09)	0.570	2 (18.18)	0.434	0 (0)	0.636	0 (0)	0.014	0 (0)	0.014	3 (27.27)	0.457
Nasal	6	6 (100)		0 (0)		3 (50)		1 (16.67)		2 (33.33)		0 (0)		1 (16.67)		1 (16.67)		2 (33.33)	
Rectal	39	39 (100)		4 (10.26)		18 (46.15)		2 (5.13)		5 (12.82)		2 (5.13)		0 (0)		0 (0)		6 (15.38)	

N: Number of isolates tested; n: Number of resistant isolates; %: Prevalence.

**Fig. 3.**
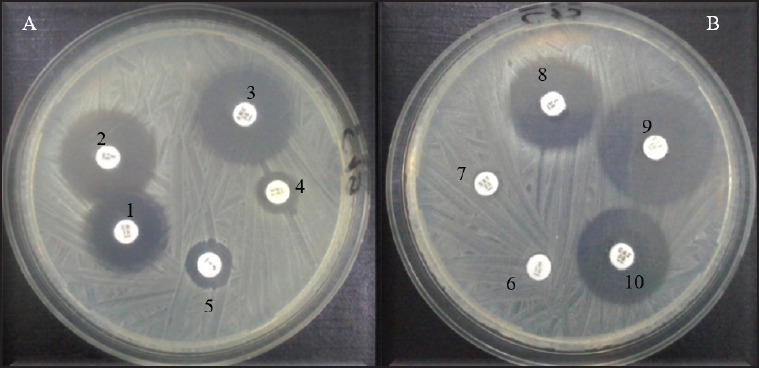
Figure 3. Antibiotic susceptibility profile of **S*. *aureus** strains. (A) 1: gentamicin (CN), 2: erythromycin (E), 3: amoxicillineclavulanic acid (AMC), 4: tetracycline (TE), 5: penicillin G (P), (B) 6: streptomycin (S), 7: cotrimoxazole (SXT), 8: chloramphenicol (C), 9: cefotaxime (CTX), 10: ceftazidime (CAZ).

Concerning *S*. *aureus* isolates, the prevalence of those resistant to penicillin (81.48%) was higher than that of isolates resistant to tetracycline (59.26%), streptomycin (22.22%), ceftazidime (22.22%), erythromycin (18.52%), chloramphenicol (14.81%), cotrimoxazole (11.11%), gentamicin (3.70%), and cefotaxime (3.70%) (Fig. 3). Unlike the prevalence of tetracycline-resistant *S*. *aureus*, which was significantly greater in Abomey-Calavi than in Cotonou (*p* > 0.05), the prevalence of penicillin-, gentamicin-, streptomycin-, erythromycin-, chloramphenicol-, ceftazidime-, cefotaxime-, and cotrimoxazole-resistant *S*. *aureus* did not vary significantly according to the variables studied (*p* > 0.05) (Table 7).

**Table 7.** Table 7. Prevalence of resistant **S*. *aureus** strains according to the variables.

Variables	N	Penicillin G		Tetracycline		Gentamicin		Streptomycin		Erythromycin		Chloramphenicol		Ceftazidime		Cefotaxime		Cotrimoxazole	
		*n* (%)	*p*-value	*n* (%)	*p*-value	*n* (%)	*p*-value	*n* (%)	*p*-value	*n* (%)	*p*-value	*n* (%)	*p*-value	*n* (%)	*p*-value	*n* (%)	*p*-value	*n* (%)	*p*-value
Municipalities																			
Abomey Calavi	15	12 (80)	0.824	12 (80)	0.014	0 (0)	0.254	3 (20)	0.756	4 (26.67)	0.223	3 (20)	0.396	2 (13.33)	0.214	0 (0)	0.254	1 (6.67)	0.411
Cotonou	12	10 (83.33)		4 (33.33)		1 (8.33)		3 (25)		1 (8.33)		1 (8.33)		4 (33.33)		1 (8.33)		2 (16.67)	
Sex																			
Male	12	8 (66.67)	0.076	8 (66.67)	0.483	0 (0)	0.362	2 (16.67)	0.534	2 (16.67)	0.824	1 (8.33)	0.396	2 (16.67)	0.534	1 (8.33)	0.254	2 (16.67)	0.411
Female	15	14 (93.33)		8 (53.33)		1 (6.67)		4 (26.67)		3 (20)		3 (20)		4 (26.67)		0 (0)		1 (6.67)	
Age																			
< 3	8	7 (87.5)	0.601	5 (62.5)	0.824	0 (0)	0.508	1 (12.5)	0.430	0 (0)	0.108	1 (12.5)	0.826	2 (25)	0.821	0 (0)	0.508	0 (0)	0.233
> 3	19	15 (78.95)		11 (57.89)		1 (5.26)		5 (26.31)		5 (26.31)		3 (15.79)		4 (21.05)		1 (5.26)		3 (15.79)	
Breed																			
Local	3	3 (100)	0.282	2 (66.67)	0.387	0 (0)	0.813	0 (0)	0.651	0 (0)	0.476	0 (0)	0.862	1 (33.33)	0.286	1 (33.33)	0.216	0 (0)	0.696
Mixed breed	7	5(71.43)		5(71.43)		1(14.28)		2(28.57)		1(14.28)		1(14.28)		1(14.28)		0 (0)		1(14.28)	
German shepherds	7	6(85.71)		5(71.43)		0 (0)		3(42.86)		3(42.86)		2(28.57)		0 (0)		0 (0)		2(28.57)	
Swiss shepherds	1	1 (100)		0 (0)		0 (0)		0 (0)		0 (0)		0 (0)		0 (0)		0 (0)		0 (0)	
Caucasian shepherds	–	–		–		–		–		–		–		–		–		–	
Malinois shepherds	–	–		–		–		–		–		–		–		–		–	
Boerbulls	3	2(66.67)		2(66.67)		0 (0)		0 (0)		1(33.33)		0 (0)		1(33.33)		0 (0)		0 (0)	
Rottweilers	–	–		–		–		–		–		–		–		–		–	
Poodles	5	5 (100)		1 (20)		0 (0)		1 (20)		0 (0)		1 (20)		3 (60)		0 (0)		0 (0)	
Bichons	1	0 (0)		1 (0)		0 (0)		0 (0)		0 (0)		0 (0)		0 (0)		0 (0)		0 (0)	
Health status																			
Apparently healthy	25	21 (84)	0.233	15 (60)	0.781	1 (4)	0.773	5 (20)	0.326	5 (20)	0.483	4 (16)	0.539	6 (24)	0.432	1 (4)	0.773	2 (8)	0.068
Sick	2	1 (50)		1 (50)		0 (0)		1 (50)		0 (0)		0 (0)		0 (0)		0 (0)		1 (50)	
Housing mode																			
In cage	19	14(73.68)	0.108	12(63.16)	0.525	0 (0)	0.116	4(21.05)	0.821	5(26,31)	0.108	3(15.79)	0.826	4(21.05)	0.821	1(5.26)	0.508	3(15.79)	0.233
Free-ranging	8	8 (100)		4 (50)		1(12.5)		2 (25)		0 (0)		1(12.5)		2 (25)		0 (0)		0 (0)	
Antibiotic therapy																			
At least once	25	20 (80)	0.483	14 (56)	0.223	1 (4)	0.773	6 (24)	0.432	5 (20)	0.483	4 (16)	0.539	6 (24)	0.432	1 (4)	0.773	3 (12)	0.603
Never	2	2 (100)		2 (100)		0 (0)		0 (0)		0 (0)		0 (0)		0 (0)		0 (0)		0 (0)	
Sample types																			
Buccal	9	7(77.78)	0.916	5(55.56)	0.960	0 (0)	0.571	3(33.33)	0.354	2(22.22)	0.497	2(22.22)	0.531	2(22.22)	0.531	1(11.11)	0.354	1(11.11)	0.648
Nasal	13	11(84.61)		8(61.54)		1(7.69)		3(23.08)		3(23.08)		2(15.38)		2(15.38)		0 (0)		2(15.38)	
Rectal	5	4 (80)		3 (60)		0 (0)		0 (0)		0 (0)		0 (0)		2 (40)		0 (0)		0 (0)	

N: Total number of isolates tested; n: Number of resistant isolates; %: Prevalence.

Furthermore, the prevalence of *S*. *aureus* MDR isolates (33.33%) was higher than that of *E*. *coli* MDR isolates (28.57%). Nevertheless, the prevalence of multidrug-resistant *E*. *coli* and *S*. *aureus* isolates did not vary according to the variables studied (Table 8).

**Table 8.** Table 8. Prevalence of multidrug-resistant **E*. *coli** and **S*. *aureus** strains according to the variables.

Variables	*E*. *coli*			*S*. *aureus*		
	*n*	*n* (%)	*p*-value	*n*	*n* (%)	*p*-value
	56	16 (28.57)	–	27	9 (33,33)	–
Municipalities						
Abomey-Calavi	34	9 (26.47)	0.665	15	5 (33.33)	1
Cotonou	22	7 (31.82)		12	4 (33.33)	
Sex						
Male	34	10 (29.41)	0.863	12	3 (25)	0.411
Female	22	6 (27.27)		15	6 (40)	
Age						
< 3	12	4 (33.33)	0.680	8	1 (12.50)	0.136
> 3	44	12 (27.27)		19	8 (42.10)	
Breed						
Local	4	1 (25)	0.263	3	0 (0)	0.547
Mixed-breed	6	4 (66.67)		7	3 (42.86)	
German shepherds	25	5 (20)		7	4 (57.14)	
Swiss shepherds	1	0 (0)		1	0 (0)	
Caucasian shepherds	2	0 (0)		–	–	
Boerbulls	8	4 (50)		3	1 (33.33)	
Rottweilers	2	1 (50)		–	–	
Poodles	5	1 (20)		5	1 (20)	
Bichons	3	0 (0)		1	0 (0)	
Health status						
Apparently healthy	50	13 (26)	0.219	25	8 (32)	0.603
Sick	6	3 (50)		2	1 (50)	
Housing mode						
In cage	45	14 (31.11)	0.395	19	7 (36.84)	0.551
Free-ranging	11	2 (18.18)		8	2 (25)	
Antibiotic therapy						
At least once	52	14 (26.92)	0.325	25	9 (36)	0.299
Never	4	2 (50)		2	0 (0)	
Sample types						
Buccal	11	3 (27.27)	0.962	9	3 (33.33)	0.177
Nasal	6	2 (33.33)		13	6 (46.15)	
Rectal	39	11 (28.20)		5	0 (0)	

N: Number of isolates tested; n: Number of multidrug-resistant isolates; %: Prevalence.

### Prevalence of resistance genes detected in antibiotic-resistant zoonotic bacteria

**Fig. 4.**
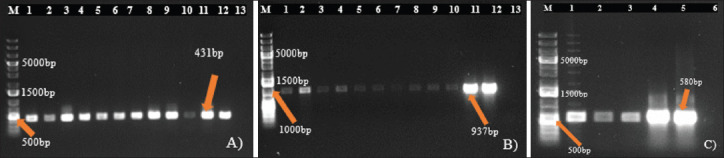
Figure 4. Agarose gel electrophoresis of amplified products for resistance genes in **E*. *coli** (A) Gene *bla*TEM (431bp): M: Molecular ladder; 1-12: Positive samples; 13: Negative control; B) Gene *tetA* (937 bp): M: Molecular ladder; 1-12: Positive samples; 13: Negative control; C) Gene *strA-strB* (580 bp); M: Molecular ladder; 1-5: Positive samples; 6: Negative control.

**Fig. 5.**
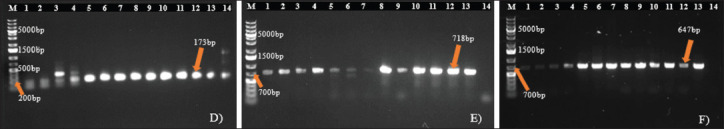
Figure 5. Agarose gel electrophoresis of amplified products for resistance genes in **S*. *aureus** (D) Gene *blaZ* (173 bp): M: Molecular ladder; 1-14: Positive samples; (E) Gene *tetK* (718 bp): M: Molecular ladder; 1-13: Positive samples; 14: Negative control; (F) Gene *tetM* (647 bp): M: Molecular ladder; 1-13: Positive samples; 14: Negative control.

Table 9 shows the resistance gene prevalence detected in *E*. *coli* and *S*. *aureus* isolates from dogs. The genes *blaTEM* (Fig. 4A), *tetA* (Fig. 4B), and *strA-strB* (Fig. 4C) were detected in 63.46% of penicillin-resistant *E*. *coli*, 62.50% of tetracycline-resistant *E*. *coli*, and 55.56% of streptomycin-resistant *E*. *coli* isolates, respectively, while the gene *tetB* was absent in all *E*. *coli* isolates. In *S*. *aureus* isolates, the genes *tetK* (Fig. 5D) and *tetM* (Fig. 5E) were detected in all tetracycline-resistant isolates investigated, whereas *blaZ* was present in 82.61% of penicillin-resistant isolates (Fig. 5F), and the genes *mecA* and *strA-strB* were absent. However, the resistance genes’ prevalence detected in *E*. *coli* and *S*. *aureus* isolates did not vary significantly according to the variables studied (Tables 10 and 11).

**Table 9.** Table 9. Prevalence of resistance genes detected in antibioticresistant **E*. *coli** and **S*. *aureus** isolates.

Resistance genes	**E*. *coli**		**S*. *aureus**	
	*n*	*n* (%)	*n*	*n* (%)
*blaTEM*	52	33 (63.46)	–	–
*blaZ*	–	–	20	19 (82.61)
*tetA*	24	15 (62.50)	–	–
*tetB*	24	0 (0.00)	–	–
*tetK*	–	–	14	14 (100)
*tetM*	–	–	14	14 (100)
*strA-strB*	9	5 (55,56)	4	0 (0.00)
*mecA*	–	–	24	0 (0.00)

N: Number of strains tested; n : Number of strains harboring the gene investigated; %: Prevalence.

**Table 10.** Table 10. Prevalence of resistance genes detected in **E*. *coli** strains according to the variables.

Variables	*blaTEM*			*tetA*			*strA-strB*		
	*n*	*n* (%)	*p*-value	*n*	*n* (%)	*p*-value	*n*	*n* (%)	*p*-value
Municipalities	52	33 (63.46)	–	24	15 (62.50)		9	5 (55.56)	
Abomey-Calavi	32	21 (65.63)	0.681	15	11 (73.33)	0.157	5	4 (80)	0.098
Cotonou	20	12 (60)		9	4 (44.44)		4	1 (25)	
Sex									
Male	31	17 (54.84)	0.117	14	9 (64.28)	0.830	6	3 (50)	0.635
Female	21	16 (76.19)		10	6 (60)		3	2 (66.67)	
Age									
< 3	11	9 (81.82)	0.154	7	4 (57.14)	0.727	2	1 (50)	0.857
> 3	41	24 (58.54)		17	11 (64.70)		7	4 (57.14)	
Breed									
Local	4	3 (75)	0.377	2	2 (100)	0.368	1	0 (0)	0.308
Mixed-breed	5	5 (100)		5	4 (80)		3	2 (66.67)	
German shepherds	23	12 (52.17)		8	4 (50)		3	1 (33.33)	
Caucasian shepherds	1	1 (100)		–	–		–	–	
Swiss shepherds	1	0 (0)		–	–		–	–	
Boerbulls	8	5 (62.50)		5	4 (80)		2	2 (100)	
Rottweilers	2	1 (50)		1	0 (0)		–	–	
Poodles	5	3 (60)		1	0 (0)		–	–	
Bichons	3	3 (100)		2	1 (50)		–	–	
Health status									
Apparently healthy	46	29 (63.04)	0.862	21	13 (61.90)	0.873	8	4 (50)	0.342
Sick	6	4 (66.67)		3	2 (66.67)		1	1 (100)	
Housing mode									
In cage	42	28 (66.67)	0.325	20	13 (65)	0.571	8	5 (62.50)	0.235
Free-ranging	10	5 (50)		4	2 (50)		1	0 (0)	
Antibiotic therapy									
At least once	49	31 (63.26)	0.905	21	14 (66.67)	0.264	8	5 (62.50)	0.235
Never	3	2 (66.67)		3	2 (33.33)		1	0 (0)	
Sample types									
Buccal	11	6 (54.54)	0.786	4	2 (50)	0.850	2	1 (50)	0.956
Nasal	6	4 (66.67)		3	2 (66.67)		2	1 (50)	
Rectal	35	23 (65.71)		17	11 (64.70)		5	3 (60)	

N: Number of strains tested; n : Number of strains harboring the gene investigated; %: Prevalence.

**Table 11.** Table 11 Prevalence of resistance genes detected in **S*. *aureus** strains according to the variables.

Variables	blaZ			tetK			tetM			strA-strB		
	*n*	*n* (%)	*p*-value	*n*	*n* (%)	*p*-value	*n*	*n* (%)	*p*-value	*n*	*n* (%)	*p*-value
Municipalities	20	19 (82.61)	0.353	14	14 (100)	–	14	14 (100)	–	4	0 (0)	–
Abomey-Calavi	11	10 (90.91)		10	10 (100)		10	10 (100)		2	0 (0)	
Cotonou	9	9 (100)		4	4 (100)		4	4 (100)		2	0 (0)	
Sex			0.209			–			–			–
Male	8	7 (87.5)		7	7 (100)		7	7 (100)		2	0 (0)	
Female	12	12 (100)		7	7 (100)		7	7 (100)		2	0 (0)	
Age			0.162			–			–			–
< 3	7	6 (85.71)		5	5 (100)		5	5 (100)		1	0 (0)	
> 3	13	13 (100)		9	9 (100)		9	9 (100)		3	0 (0)	
Breed			0.676		–			–			–	
Local	3	3 (100)		2		2 (100)	2		2 (100)	–		–
Mixed-breed	4	4 (100)		4		4 (100)	4		4 (100)	1		0 (0)
German shepherds	5	5 (100)		4		4 (100)	4		4 (100)	2		0 (0)
Swiss shepherds	1	1 (100)		–		–	–		–	–		–
Boerbulls	2	2 (100)		2		2 (100)	2		2 (100)	–		–
Poodles	5	4 (80)		1		1 (100)	1		1 (100)	1		0 (0)
Bichons	–	–		1		1 (100)	1		1 (100)	–		–
Health status			0.814			–			–			–
Apparently healthy	19	18 (94.74)		13	13 (100)		13	13 (100)		3	0 (0)	
Sick	1	1 (100)		1	1 (100)		1	1 (100)		1	0 (0)	
Housing mode			0.451			–			–			–
In cage	13	12 (92.31)		10	10 (100)		10	10 (100)		3	0 (0)	
Free-ranging	7	7 (100)		4	4 (100)		4	4 (100)		1	0 (0)	
Antibiotic therapy			0.732			–			–			–
At least once	18	17 (94.44)		12	12 (100)		12	12 (100)		4	0 (0)	
Never	2	2 (100)		2	2 (100)		2	2 (100)		–	–	
Sample types			0.293			–			–			–
Buccal	6	5 (83.33)		3	3 (100)		3	3 (100)		2	0 (0)	
Nasal	10	10 (100)		8	8 (100)		8	8 (100)		2	0 (0)	
Rectal	4	4 (100)		3	3 (100)		3	3 (100)		–	–	

N: Number of strains tested; n : Number of strains harboring the gene investigated; %: Prevalence.

### Prevalence of virulence genes detected in antibiotic-resistant zoonotic bacteria

**Fig. 6.**
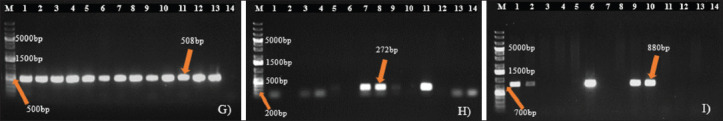
Figure 6. Agarose gel electrophoresis of amplified products for virulence genes in **E*. *coli** (G) Gene *fimH* (508 bp): M: Molecular ladder; 1-13: Positive samples; 14: Negative control; (H) Gene *kpsMT II* (272 bp): M: Molecular ladder; 5,7-11: Positive samples; 1-4,6,12,13: Negative samples; 14: Negatixve control; I) Gene *fyuA* (880 bp): M: Molecular ladder; 1,2,6,9,10: Positive samples; 3-5,7,8,11-13: Negative samples; 14: Negative control.

**Fig. 7.**
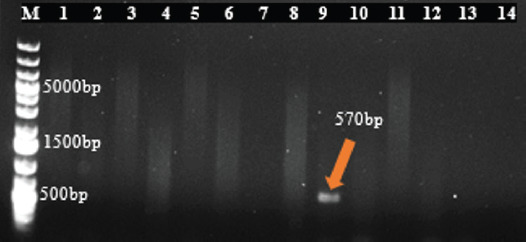
Figure 7. Agarose gel electrophoresis of amplified products for gene eae in **E*. *coli** (570 bp) M: Molecular ladder; 9: Positive sample; 1-8,10-13: Negative samples; 14: Negative control.

Table 12 shows the prevalence of virulence genes investigated in *E*. *coli* and *S*. *aureus* isolates. The table analysis reveals a high prevalence of the gene *fimH* (61.54%) (Fig. 6G) detected in the isolates, followed respectively by that of the genes *kpsMTII* (26.92%) (Fig. 6H), *fyuA* (19.23%) (Fig. 6I), and *eae* (1.92%) (Fig. 7). Only the prevalence of the gene *eae* varied significantly according to the breeds (*p* < 0.05) (Table 13). Furthermore, the prevalence of fnbA varied significantly according to the municipality of the dogs (*p* < 0.05), whereas the prevalence of pvl did not vary significantly according to the variables studied (*p* > 0.05). (Table 14).

**Table 12.** Prevalence of virulence genes detected in **E*. *coli** and **S*. *aureus** isolates.

Virulence genes	*n*	*n* (%)
*Escherichia coli*		
*stx1*	52	0 (0.00)
*stx2*	52	0 (0.00)
*fimH*	52	32 (61.54)
*eae*	52	1 (1.92)
*kpsMTII*	52	14 (26.92)
*fyuA*	52	10 (19.23)
*Staphylococcus aureus*		
*pvl*	24	5 (20.83)
*sea*	24	0 (0.00)
*seb*	24	0 (0.00)
*sec*	24	0 (0.00)
*hla*	24	0 (0.00)
*hlb*	24	0 (0.00)
*eta*	24	0 (0.00)
*etb*	24	0 (0.00)
*fnbA*	24	5 (20.83)
*fnbB*	24	0 (0.00)

N: Number of strains tested; n: Number of strains harboring the gene investigated; %: Prevalence.

**Table 13.** Table 13. Prevalence of virulence genes detected in **E*. *coli** strains according to the variables.

Variables	*N*	*fimH*		*eae*		*kpsMT-II*		*fyuA*	
		*n* (%)	*p*-value	*n* (%)	*p*-value	*n* (%)	*p*-value	*n* (%)	*p*-value
Municipalities	52	32 (61.54)		1 (1.92)		14 (26.92)		10 (19.23)	
Abomey-Calavi	32	22 (68.75)	0.176	1 (3.12)	0.425	7 (21.87)	0.299	6 (18.75)	0.911
Cotonou	20	10 (50)		0 (0)		7 (35)		4 (20)	
Sex									
Male	31	17 (54.84)	0.228	1 (3.23)	0.406	7 (22.58)	0.391	5 (16.13)	0.490
Female	21	15 (71.43)		0 (0)		7 (33.33)		5 (23.81)	
Age									
< 3	11	8 (72.73)	0.390	0 (0)	0.601	2 (18.18)	0.462	3 (27.27)	0.446
> 3	41	24 (58.54)		1 (2.43)		12 (29.27)		7 (17.07)	
Breed									
Local	4	2 (50)	0.066	0 (0)	0.001	0 (0)	0.125	1 (25)	0.185
Mixed-breed	5	4 (80)		0 (0)		1 (20)		1 (20)	
German shepherds	23	12 (52.17)		0 (0)		7 (30.43)		4 (17.39)	
Swiss shepherds	1	0 (0)		0 (0)		0 (0)		0 (0)	
Caucasian shepherds	1	1 (100)		0 (0)		1 (100)		0 (0)	
Boerbulls	8	8 (100)		0 (0)		1 (12.50)		0 (0)	
Rottweilers	2	1 (50)		1 (50)		2 (100)		2 (100)	
Poodles	5	1 (20)		0 (0)		2 (40)		1 (20)	
Bichons	3	3 (100)		0 (0)		0 (0)		1 (33.33)	
Health status									
Apparently healthy	46	28 (60.87)	0.784	1 (2.17)	0.715	13 (28.26)	0.547	9 (19.56)	0.865
Sick	6	4 (66.67)		0 (0)		1 (16.67)		1 (16.67)	
Housing mode									
In cage	42	25 (59.52)	0.540	1 (2.38)		11 (26.19)	0.807	7 (16.67)	0.336
Free-ranging	10	7 (70)		0 (0)		3 (30)		3 (30)	
Antibiotic therapy									
At least once	49	30 (61.22)	0.851	1 (2.04)	0.803	13 (26.53)	0.796	9 (18.37)	0.523
Never	3	2 (66.67)		0 (0)		1 (33.33)		1 (33.33)	
Sample types									
Buccal	11	8 (72.73)	0.277	0 (0)	0.781	1 (9.09)	0.322	3 (27.27)	0.748
Nasal	6	5 (83.33)		0 (0)		2 (33.33)		1 (16.67)	
Rectal	35	19 (54.28)		1 (2.86)		11 (31.43)		6 (17.14)	

N: Number of strains tested; n : Number of strains harboring the gene investigated; %: Prevalence.

**Table 14.** Table 14. Prevalence of virulence genes detected in **S*. *aureus** strains according to the variables.

Variables	*N*	*pvl*		*fnbA*	
		*n* (%)	*p*-value	*n* (%)	*p*-value
Municipalities	24	5 (20.83)	–	5 (20.83)	
Abomey-Calavi	13	1 (7.69)	0.085	5 (38.46)	0.021
Cotonou	11	4 (36.36)		0 (0)	
Sex					
Male	11	1 (9.09)	0.193	3 (27.27)	0.475
Female	13	4 (30.77)		2 (15.38)	
Age					
< 3	8	1 (1.25)	0.477	1 (12.5)	0.477
> 3	16	4 (25)	4 (25)		
Breed					
Local	3	1 (33.33)	0.732	1 (33.33)	0.319
Mixed-breed	5	0 (0)		1 (20)	
German shepherds	6	1 (16.67)		2 (33.33)	
Swiss shepherds	1	0 (0)		1 (100)	
Boerbulls	3	1 (33.33)		0 (0)	
Poodles	5	2 (40)		0 (0)	
Bichons	1	0 (0)		0 (0)	
Health status					
Apparently healthy	22	5 (22.73)	0.449	5 (22.73)	0.448
Sick	2	0 (0)		0 (0)	
Housing mode					
In cage	17	4 (23.53)	0.612	2 (11.76)	0.088
Free-ranging	7	1 (14.28)		3 (42.86)	
Antibiotic therapy					
At least once	22	5 (22.73)	0.449	5 (22.73)	0.449
Never	2	0 (0)		0 (0)	
Sample types					
Buccal	7	1 (14.28)	0.491	1 (14.28)	0.491
Nasal	12	2 (16.67)		2 (16.67)	
Rectal	5	2 (40)		2 (40)	

N: Number of strains tested; n: Number of strains harboring the gene investigated; %: Prevalence.

**Fig. 8.**
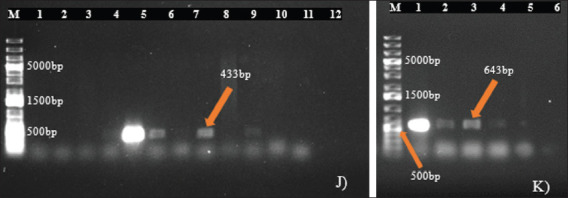
Figure 8. Agarose gel electrophoresis of amplified products for virulence genes in **S*. *aureus** (J) Gene pvl (433 bp): M: Molecular ladder; 4-6,8,10: Positive samples; 1-3,7,9,11: Negative samples; 12: Negative control; K) Gene fnbA (643 bp): M: Molecular ladder; 1-5: Positive samples; 6: Negative control

For *S*. *aureus* strains, only the genes *pvl* (Fig. 8J) and *fnbA* (Fig. 8K) were detected, with a prevalence of 20.83% for each gene. The genes *sea*, *seb*, *sec*, *hla*, *hlb*, *eta*, *etb*, and *fnbB* were not present in the isolates.

### Partial genome sequencing of antibiotic-resistant zoonotic bacteria

**Fig. 9.**
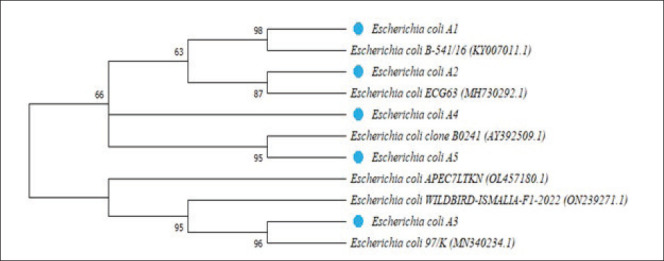
Figure 9. Phylogenetic tree of the *fimH* gene of **E*. *coli** isolates and **E*. *coli** strains retrieved from GenBank. Blue-chipped strains are from this study, while non-chipped strains are from GenBank retrievals.

The analysis of Figure 9 shows a strong similarity of *Escherichia coli* A1 with *E*. *coli* B-541/16 (KY007011.1) isolated from liquor in Russia; *E*. *coli* A2 with *E*. *coli* ECG63 (MH730292.1) isolated from humans in Spain; *E*. *coli* A3 with *E*. *coli* 97/K (MN340234.1) isolated from milk in India; *E*. *coli* A5 with *E*. *coli* clone B0241 (AY392509.1) from the bovine clinical case in Belgium, and *E*. *coli* A4 with *E*. *coli* A5.

**Fig. 10.**
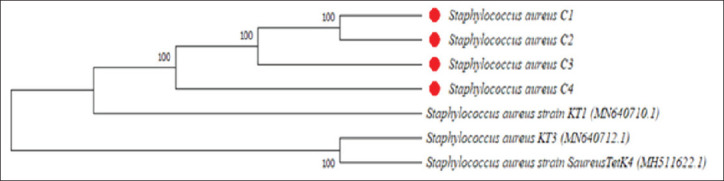
Figure 10. Phylogenetic tree of the *tetK* gene of **S*. *aureus** isolates and **S*. *aureus** strains retrieved from GenBank. Red-chipped strains are from this study, while non-chipped strains are from GenBank.

Additionally, the four **S*. *aureus** strains (C1, C2, C3, and C4) investigated in this study are closely related. They form a very close group, suggesting recent evolutionary divergence and significant genetic similarity. They show strong homology with **S*. *aureus** strain KT1 (MN640710.1) isolated from clinical samples in Pakistan (Fig. 10).

## Discussion

The present study determined the prevalence of zoonotic bacteria, including *E*. *coli* and *S*. *aureus* isolates, and their susceptibility profile to the various antibiotics currently available. Frequent human contact with reservoirs of zoonotic agents represents a danger to humans. The more frequently a zoonotic agent is found in a reservoir close to humans, the greater the risk to humans.

*Escherichia coli* is a commensal bacterium of the vertebrate gut, but some *E*. *coli* strains are responsible for various diseases, including intestinal and extra-intestinal disorders in humans and animals [2]. In the current study, the overall prevalence of *E*. *coli* recorded in dogs was moderate, 41.07%. The significant variation of *E*. *coli* prevalence according to age may be linked to the small sample size of dogs under 3 years of age compared with those over 3 years of age. Similar results were reported in Ethiopia with a significant difference between the prevalences of *E*. *coli* within age categories [19]. The prevalence of *E*. *coli* in sick dogs in our study was significantly greater than in apparently healthy dogs. The high prevalence of *E*. *coli* in sick dogs may explain their symptoms. *Escherichia coli* can be responsible for diarrhea and general signs such as loss of appetite and emaciation [2]. Furthermore, the prevalence of *E*. *coli* isolated from rectal swabs was significantly greater than that from buccal and nasal swabs, indicating that *E*. *coli* strains are more likely to be released into the environment *via* feces and may infect animals or humans if they come into contact with the contaminated biological material [20]. Different prevalences of *E*. *coli* isolated from rectal samples have also been recorded in previous studies: 24.20% in Ethiopia [19]. These variations in prevalence would be linked to the difference in geographical area, the period when the study was carried out, and the method used to isolate strains.

*Staphylococcus aureus* is among the leading infectious agents responsible for diseases and death worldwide [21]. *S*. *aureus* can cause a broad variety of diseases, ranging from moderately severe skin infections to fatal pneumonia and septicemia [21]. In this study, the prevalence of **S*. *aureus** was significantly higher in dogs that had undergone antibiotic therapy at least once compared with those that had never undergone antibiotic therapy before sampling. This high prevalence can be associated either with the fact that the *S*. *aureus* strains isolated had infected the dogs after the period of antibiotic therapy, the antibiotics used during antibiotic therapy were inadequate to fight the *S*. *aureus* strains carried by the dogs concerned, or the *S*. *aureus* strains had developed resistance against the antibiotics administered to the dogs during antibiotic therapy. There is a difference between the prevalence of *S*. *aureus* reported in previous studies and that recorded in the present study. Indeed, the prevalence of *S*. *aureus* isolated from dogs was 4.9% in Hungary [22]. These prevalences are lower than those recorded in this study, but those reported in the USA (20.6%) [23] are similar to those determined in our case. The differences in geographical area, sample size, breeds, sex, health status of dogs, type or method of sampling, and sample analysis could explain the variations in prevalence between studies.

The study also revealed the presence of *E*. *coli* and *S*. *aureus* strains resistant to certain antibiotics. The different levels of antibiotic resistance correlate with the frequency and extent of their use in various countries [12]. In the current study, the prevalence of *E*. *coli* and *S*. *aureus* strains, penicillin- and tetracycline-resistant, was higher. These results may be linked to the intensive use and misuse of these antibiotics in the two municipalities. On the other hand, the prevalence of *E*. *coli* and *S*. *aureus* strains resistant to amoxicillin-clavulanic acid, gentamicin, streptomycin, ceftazidime, and cefotaxime was low. These results confirm the low frequency of use of these antibiotics in the two municipalities. The low prevalence of erythromycin-resistant *S*. *aureus* isolates indicates that this antibiotic is less widely used in the study area. The high prevalence of penicillin- and tetracycline-resistant strains poses a health threat not only to the dogs harboring them but also to those who come into contact with them. In the event of an infection, the situation can lead to therapeutic failure, prolonged illness, weakened protection during surgery, or even the death of dogs carrying antibiotic-resistant strains. Dog owners may incur considerable expenses in treating their pets. Furthermore, individuals who come into contact with these dogs may be exposed to infections and develop diseases caused by antibiotic-resistant agents. Several studies carried out in other countries around the world revealed the susceptibility profile of these bacterial strains to antibiotics, and the MDR strains were identified. In South Africa, 99.4% of *E*. *coli* isolates were resistant to penicillin G, 28.3% to gentamicin, 24.7% to cotrimoxazole, 24.6% to chloramphenicol, and 58.3% to amoxicillin-clavulanic acid [24]. In Brazil, *E*. *coli* strains were resistant to gentamicin (30.9%), amoxicillin-clavulanic acid (35.7%), ceftazidime (16.6%), cefotaxime (19.4%), chloramphenicol (23.8%),and cotrimoxazole (30.9%) [25]. In Nigeria, ten *S*. *aureus* strains were resistant to penicillin, 50% were resistant to erythromycin and oxytetracycline, and 70% were resistant to gentamicin [26]. In Brazil, 66.67% of *S*. *aureus* strains isolated from sick dogs were resistant to penicillin, 33.33% to erythromycin and tetracycline, but no isolate was resistant to cotrimoxazole, gentamicin, and chloramphenicol [8]. The different levels of antibiotic resistance correlate with the frequency and extent of their use in various countries [12]. Our results, therefore, reveal that penicillin and tetracycline are more widely used in the two municipalities compared to ceftazidime, cefotaxime, chloramphenicol, gentamicin, and amoxicillin-clavulanic acid.

Furthermore, MDR strains are bacteria that have acquired resistance to at least one agent in three or more antimicrobial categories [18]. The prevalence of MDR *E*. *coli* and *S*. *aureus* isolates in the current research is above 28%. This result may be attributed to the intensive use of penicillin, tetracyclines, and streptomycin in dogs in our country. However, there is a difference between the results found in this study and those reported in other countries. The prevalence of MDR *E*. *coli* isolates is well above that recorded in Ethiopia [19]. These differences in results can be attributed to the difference in antibiotic use policies in each country, the difference in diagnostic methods before antibiotic application, the antibiotics available in each country or region, veterinary antibiotic import policies, and the cost of antibiotics.

The current study reveals resistance genes detected in *E*. *coli* and *S*. *aureus* isolates. Bacteria develop mechanisms of resistance to antibiotics, including the efflux pump, alteration of the drug target site, enzymatic inactivation of the antimicrobial agent, and efflux pump and sequestration of the antimicrobial agent [3]. These mechanisms are encoded by resistance genes. The presence of the gene *bla*TEM in **E*. *coli** isolates indicates that there has been a spread of bacteria that produce ESBL enzymes [27]. The high prevalence of *E*. *coli* harboring the gene *bla*TEM in our study can be explained by the intensive use of penicillin in our country. The prevalence of *bla*TEM detected in *E*. *coli* isolates in this study is above that reported in Brazil [28]. Our result suggests the necessity of an antibiotic susceptibility test before using penicillin on the dogs in the two municipalities. *tetA* and *tetB* encode the efflux pump, one of the more commonly described mediators of tetracycline resistance in Enterobacterales, including *E*. *coli* [29]. This study found *tetA* in 62.50% of *E*. *coli* strains phenotypically resistant to tetracycline. The high prevalence of *E*. *coli* harboring *tetA* can be linked to the intensive use of tetracycline in dogs, which in turn can be linked to their price.

Indeed, in Benin, and specifically in Abomey-Calavi and Cotonou, the price of oxytetracycline, a tetracycline derivative, is cheaper than other antibiotics used in dogs. This makes oxytetracycline more accessible and more frequently used in treating infections in dogs. As for *tetB*, none of the *E*. *coli* isolates carried it. These results also indicate that the gene *tetA* is the most implicated in tetracycline resistance developed by *E*. *coli* strains investigated in the municipalities. The genes *strA-strB* probably confer highlevel resistance to streptomycin [30]. The prevalence of *strA-strB* detected in *E*. *coli* strains phenotypically displaying streptomycin resistance was 55.56%. This prevalence is higher than the 0.89% reported in Australia [31]. These results reflect the variation in *strA-strB* prevalence between countries, probably linked to the policy of streptomycin use.

The gene *blaZ* encodes for beta-lactamase enzymes, which mediate penicillin resistance in *S*. *aureus* strains [3]. This study recorded a high prevalence of **S*. *aureus** harboring *bla*Z. A high prevalence of *S*. *aureus* harboring this gene had previously been reported in Portugal [32]. These results show the extent to which *S*. *aureus* strains are becoming resistant to penicillin and suggest the search for alternatives to replace this antibiotic. In this study, *tetK* and *tetM* were detected in all fourteen *S*. *aureus* strains investigated. The genes *tetK* and *tetM* encode tetracycline resistance in *S*. *aureus* strains. *tetK* encodes for the efflux pump system, whereas *tetM* encodes the protection of the ribosome [3]. These results can be attributed to the intensive use of tetracycline antibiotics such as oxytetracycline, which is frequently used in dogs in our country. This study reveals the absence of the *strA-strB* gene in all four *S*. *aureus* strains that showed streptomycin phenotypic resistance. Given that streptomycin resistance can be encoded by several genes in *S*. *aureus* strains, the phenotypic resistance recorded in this study can be associated with the uninvestigated genes. The study of these genes will enable us to discover the genes responsible for the observed resistance. The absence of *mecA* in *S*. *aureus* isolates in this study may be linked to the infrequent use of methicillin in dogs in our country.

The virulence factors are specific molecules produced and released by pathogenic agents [2]. The virulence factors are encoded by specific genes located on the chromosome or mobile genetic elements (plasmids or transposons) [2]. Several virulence genes are involved in infections caused by *E*. *coli* strains [2]. The gene *fimH* encodes the adhesin FimH, which is a colonization factor in extraintestinal infections, mediates binding to urothelium and invasion, and biofilm formation [2]. This gene is frequently detected in uropathogenic *E*. *coli* (UPEC) strains and adherent-invasive *E*. *coli* (AIEC) strains [2]. The presence and the high prevalence of *fimH* detected in the isolates in this study indicate that the strains harboring this gene may be UPEC or AIEC. Studying the pathogenesis of these strains will reveal their pathogenic powers. A high prevalence of *E*. *coli* harboring *fimH* has been reported in Iran [33]. Hojati et al. [34] postulated that *fimH* could be used as a diagnostic marker for *E*. *coli*. The results obtained from the partial sequencing of this gene in this study reinforce this hypothesis. *kpsMTII* encodes for the capsular proteins K1 or K5, enabling bacteria to escape phagocytosis [2]. *kpsMTII* can be detected in UPEC and AIEC strains [2]. In our study, *kpsMTII* was present in 26.92% of *E*. *coli* isolates, revealing that strains harboring this gene could belong to either the UPEC or AIEC category. Further studies will reveal the true category of these strains. Furthermore, it should be noted that the prevalence of strains harboring *kpsMTII* is lower than that reported in the USA [35]. These differences in results highlight the wide distribution of strains harboring *kpsMTII* and the need to take precautions against these zoonotic pathogens. *fyuA* encodes a ferric scavenger receptor, FyuA, which is involved in iron uptake and biofilm formation [2]. This gene can be detected in UPEC and AIEC strains [2].

In the present study, *fyuA* was detected in 19.23% of *E*. *coli* isolates. Compared with the prevalence reported in Japan (85.71%) [36], the prevalence of *fyuA* in our study is low. This result does not suggest neglect of the dangers posed by strains harboring this gene, but rather a search for ways to fight against these zoonotic agents to preserve the health of those who frequently come into contact with carrier dogs. *eae*, located in the locus of enterocyte effacement (LEE) pathogenicity island, encodes an outer membrane protein, intimin, an important virulence factor that plays a critical role in intestinal colonization [2]. In this study, one strain (1.92%) carried *eae*, indicating that it belonged to either the STEC, EHEC, or EPEC groups. Further studies will enable us to categorize this strain better. In previous studies, a low prevalence of *E*. *coli* strains harboring *eae* has also been reported in Brazil [28]. These results indicate that the gene *eae* is less frequent in *E*. *coli* strains isolated from dogs. The genes *stx1* and *stx2* encode the Shiga toxins 1 (*Stx1*) and 2 (*Stx2*), respectively, two major virulence factors of STEC strains, particularly EHEC strains, which cause diarrhea, hemorrhagic colitis with bloody diarrhea, and hemolytic uremic syndrome in humans and are implicated in several foodborne outbreaks in developed countries [2]. In the present study, no isolate harbored *stx1* and *stx2*. Similar results have been reported in studies conducted in different countries around the world, such as Italy [37]. These different results indicate that *E*. *coli* harboring the *stx1* and *stx2* genes are less common in dogs.

*Staphylococcus aureus* is the most pathogenic member of the genus *Staphylococcus*, which produces virulence factors encoded by virulence genes [38]. The gene *pvl* encodes PVL, which is a pore-forming cytotoxic secreted toxin [38]. In the study conducted by Jaiswal et al. [39], PVL-positive *S*. *aureus* was strongly associated with skin and soft tissue infection. In this study, the gene *pvl* was present in 20.83% of the *S*. *aureus* strains investigated. The presence of *pvl* in these strains reveals their virulence and, therefore, their ability to cause either skin and soft tissue infections or pneumonia in humans. Studying these strains’ pathogenesis will reveal their true pathogenic power. In this study, *sea*, *seb*, and *sec* genes were not detected in any *S*. *aureus* isolate. These results are in line with those reported in *S*. *aureus* strains isolated from dogs in Hungary [22]. In contrast, *sea* was detected in Japan [40], but *seb* and *sec* were not detected. These different results reveal the rarity of *S*. *aureus* strains harboring these genes in dogs. In this study, *fnbA* was detected in five *S*. *aureus* isolates. The presence of this gene had been reported in Hungary [22]. However, no isolate harbored the *fnbB* gene in this study. This result differs from those reported in Hungary [22], where *fnbB* was present in the *S*. *aureus* isolates. These differences in results may be attributed to geographical differences. In our study, the genes *hla* and *hlb* were not detected in any isolate of *S*. *aureus*. Asanin et al. [41] hadn’t detected *hla* in *S*. *aureus* strains tested in Serbia, but *hlb* was detected in four strains. In the study performed in Hungary [22], these genes were detected in MRSA strains. These differences in recorded prevalence indicate a variation in the prevalence of *hla* and *hlb* depending on the countries where the studies were carried out. In this study, the genes *eta* and *etb* were not detected in any *S*. *aureus* isolates investigated. These results are similar to those reported in the studies conducted in Hungary [22]. These results suggest that the genes *eta* and *etb* are not frequent in **S*. *aureus** isolated from dogs.

## Conclusion

The present study reveals a high prevalence of MDR isolates, mostly resistant to penicillin, tetracycline, and streptomycin, which are commonly used in dogs in Abomey-Calavi and Cotonou municipalities in southern Benin. Penicillin, tetracycline, and streptomycin resistance genes were detected in retrieved *E*. *coli* and *S*. *aureus* isolates. Furthermore, *E*. *coli* and *S*. *aureus* isolates harbor virulence genes, indicating the potential pathogenicity of the isolates. This study reveals the importance of laboratory diagnosis before any use of antibiotics in dogs to avoid therapeutic failures; the need to observe hygienic practices to avoid exchanges of these zoonotic agents between humans and the dogs carrying them; and the urgency of searching for alternatives to replace antibiotics against MDR.

## List of abbreviations

°C, degree Celsius

AMR, antimicrobial resistance

DNA, deoxyribonucleic acid

EMB, Eosin Methylene Blue

h, hours

MDR, multidrug-resistant

min

minutes

ml, milliliter

PCI, Phenol-Chloroform-Isoamyl

PCR, polymerase chain reaction

SDS, sodium dodecyl sulfate

sec, seconds

SIM, sulphide-indole-motility

μl, microliter.
